# Further Questions and Answers

**Published:** 1917-02-10

**Authors:** 


### THE COLLEGE AND IRELAND.

### Further Questions and Answers.

We have received the following replies to a number of questions put at the Dublin meeting on January 27 which were unanswered for want of time.

### State Registration and the College.

*Q.* When State Registration is an accomplished fact, will a fully trained nurse be able to get her State Registration without becoming a member of the College?

*A.* Yes. The certificate granted by the State will probably entitle the holder to partake of the privileges of the College, but it is not anticipated that any compulsion in this respect would be exercised.

*Q.* Suppose a member wishes to withdraw from membership of the College, how would she be affected as regards her State Registration? Would she still retain her State Registration?

*A.* A certificate granted by the State would be granted for all time, and could only be cancelled by the Disciplinary Council called into existence by the State to regulate these matters.

### Some Misapprehensions Dealt With.

*Q.* Does the College of Nursing consider that the work of the trained nurse is not of sufficient importance to the State to entitle her to a certificate issued solely on her behalf, and separate and distinct from "other branches of women's work connected with hospitals"? (See Draft Supplemental Charter A.)

*Q.* If the same authority proposes to grant certificates to various workers—to those trained in special work only, and also to the partially trained women—does it not greatly lessen the value of the trained nurses' certificate? (See Supplemental Charter, Draft C.)

*A.* It is because the College of Nursing considers it of such grave importance that the fully trained woman should be hall-marked that it has been founded and is placing State Registration as its first object. It may be pointed out further that only the fully trained woman is entitled to be registered as a member of the College. This privilege will not be granted to "other branches of women's work connected with hospitals," and this has been emphatically stated over and over again.

(The second question is answered above.)

*Q.* Considering the thousands of trained nurses working on their own account, why have they no direct representation on the Council?

*A.* This question appears to be answered in the reply above.

### Special Meetings of the College.

*Q.* Why has it been made difficult for members of the College to obtain a special meeting? In Bye-law 8, the necessary requisition for a special meeting must be signed by at least 100 members of the Corporation, and by not less than one-fourth of the members of the Council who are then entitled to be present and vote at such meeting. According to this bye-law, very little power is given to members for redress; whereas the President and Council can hold a meeting whenever they choose.

*A.* This questioner seems to have overlooked the fact that the Register will soon be a matter of thousands of members. One hundred, therefore, would appear to be a quite reasonable proportion. It is no more desirable that a meeting should be too easily summoned than that, on the other hand, there should be undue difficulty.

### The Question of Democratic Control.

*Q.* Why are all the hon. officers of the College men, and why were the deputations of the Medico - Psychological Society, the Association of Poor-Law Guardians, and the British Medical Association received by the officers only with not one matron or nurse present? This does not seem to me to be democratic control by *Nurses?*

*A.* Because these deputations had desired to be received by the Chairman of the Council of the College of Nursing. It is pointed out further that the Chairman in all instances acted in co-operation with, and also with the authority of his Council. The hon. officers were appointed by the desire of the Council, and were specially selected for their business capacity, and sympathetic attitude towards nursing matters.

### Nurses' Leagues and Representation.

*Q.* There are large numbers of Nurses' Associations and Leagues throughout the United Kingdom. Why were none of these bodies asked to elect representatives to act on the Council of the College of Nursing?

*A.* One of the great objects of the College of Nursing in its foundation was to establish a Register of Trained Nurses which would become a reliable electorate from which direct representation of the Nursing Profession could be gained. The compiling of this Register is rapidly proceeding, and a very large, representative, and absolutely reliable electorate will be in existence, and will at once proceed to accept responsibility for the direct representation of the nurses.

[*Note.* — An increasing number of members of the Nurses' Leagues of hospitals having nurse-training schools are joining the Register every day.]

### The Most Autocratic Proposal Ever Made.

*Q.* This movement is spoken of as a *democratic one.* Would you please tell us when it will become *democratic;* up to the present it seems to be highly *autocratic,* trained nurses having been ignored?

*A.* Trained nurses have never been ignored in this matter. From the formation of the first Council at least two-thirds of the members have been trained nurses in active work, and in close touch not only with nursing education, but also with the certificated nurse. In the year 1918, and every successive year, one-third of the Council as at present constituted retires, the vacancy being filled by the postal vote of the electorate, consisting of the nurses registered on the College Register. No more democratic proposal has previously been made in connection with the organisation of the profession.

(Signed) E. COX-DAVIES.

M. S. RUNDLE.

